# The Influence of Nutrition on Intestinal Permeability and the Microbiome in Health and Disease

**DOI:** 10.3389/fnut.2022.718710

**Published:** 2022-04-25

**Authors:** Orsolya Inczefi, Péter Bacsur, Tamás Resál, Csilla Keresztes, Tamás Molnár

**Affiliations:** ^1^Department of Gastroenterology, Albert Szent-Györgyi Medical School, University of Szeged, Szeged, Hungary; ^2^Department for Medical Communication and Translation Studies, Albert Szent-Györgyi Medical School, University of Szeged, Szeged, Hungary

**Keywords:** intestine, permeability, microbiome, nutrition, disease, leaky gut, intestinal barrier, gut microbiota

## Abstract

The leakage of the intestinal barrier and the disruption of the gut microbiome are increasingly recognized as key factors in different pathophysiological conditions, such as irritable bowel syndrome (IBS), inflammatory bowel disease (IBD), chronic liver diseases, obesity, diabetes mellitus, types of cancer, and neuropsychiatric disorders. In this study, the mechanisms leading to dysbiosis and “leaky gut” are reviewed, and a short summary of the current knowledge regarding different diseases is provided. The simplest way to restore intestinal permeability and the microbiota could be ideal nutrition. Further therapeutic options are also available, such as the administration of probiotics or postbiotics or fecal microbiota transplantation.

## Introduction

The intestinal epithelial barrier (IEB) has the greatest surface in the body, that separates the interior part from the environment. The IEB has two main roles: it is both responsible for nutrient absorption and serves as the first line of defense against external pathogens. The IEB has a well-regulated structure. Its permeability is organized into transcellular and paracellular ways. Recently, the importance of the mucous surface in the intestine has also been recognized as a crucial regulator of permeability. The mucous surface is colonized by the commensal microbiota, which helps in nutrient production, the elimination of pathogens, and the overall maintenance of gut health. Food passing through the gastrointestinal tract has an enormous influence on the microenvironment of the intestinal microbiome and also on the IEB. Food can be a selective advantage for certain members of the microbiota that have influence on gut function. In conditions with increased intestinal permeability, the alteration of the microbiota is also often observed. In the last decade, there was a great effort made in the scientific community to verify the direct link between the microbiota and disease progression, and the regulatory options of these diseases through the microbiota. In this review, the relations based on the currently available literature were explored. The impact of food intake on gut microbiota, as well as the mechanism of how it modifies intestinal permeability, which results in various pathological conditions, is described.

## Intestinal Permeability and the Microbiome

The intestinal microbiota is a complex but not fully understood ecosystem of microorganisms. Recent signs of progress in molecular biology techniques have made it possible to collect more data about the impact of the microbiota (1). Usually, the microbiota consists of mostly anaerobic bacteria, fungi, archaea, viruses, phages, and protozoa, which live in balance with the host organism. This genome contains 100 times as many genes as the human host (2, 3). The microbiota can be considered an “organ” within the body, as it has a complex structure and specific functions (4).

### Microbiome and Microbiota Composition

Disruption in the balance of the bacterial composition of the microbiota is referred to as dysbacteriosis (5). A very important aspect of microbiota analysis is that the “normal microbiota composition” has not yet been clearly defined. Right after birth, the microbiota is present in the human body, and there occur changes in its composition during the first years of development, which continue until the beginning of aging in an individual (6, 7). Aging induces moderate changes in the microbiome, wherein diversity is diminished in old people, especially in people living in older adult homes. The composition of the microbiota also depends on the host’s genetics, diet, and cultural and geographical factors, which make it even more difficult to identify an ideal, universal profile of the healthy microbiome (8, 9). Furthermore, lifestyle can significantly influence the microbial community; consequently, different microbiomes and microbiota are present in healthy rural and urban people (10–13). Due to this high variability, no conclusions should be drawn about the healthy microbiome and microbiota composition based on taxonomic classification, and as a result, more useful data could be provided by the metabolic functions (14–16).

### Intercellular Junctions and Intestinal Epithelial Barrier Permeability

**FIGURE 1:**
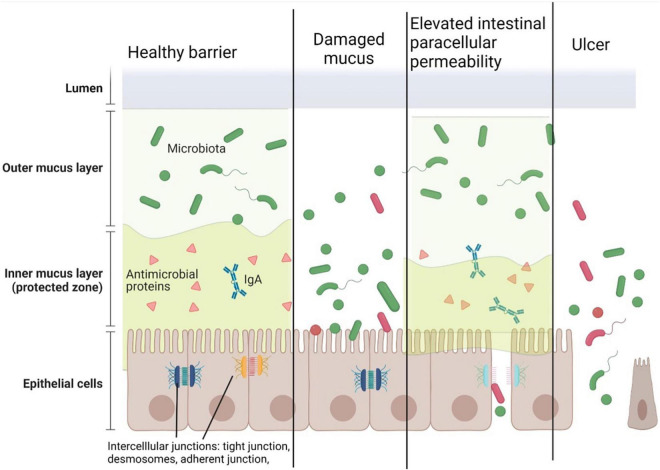
Components of the healthy intestinal barrier and the potential mechanisms of barrier damage. The intestinal epithelial barrier consists of outer and inner layers of mucus, epithelial cells, and intercellular junctions. In a healthy stage, the outer mucous layer forms a 3-dimensional network in the gut lumen containing microbiota. The inner mucous layer containing antimicrobial peptides and secretory IgA keeps away the microbes from the epithelial cells. Intercellular junctions (tight junctions, adherent junctions, and desmosomes) connect the cells to form a barrier between the subepithelial surface and the microbiota.

The regulation of IEB permeability is a complex, not clearly understood system. Intercellular junctions, such as tight junction, adherent junction, and desmosomes, have a key role in the paracellular passage (17, 18). The subunits of these subcellular structures are located on the lateral membranes of the epithelial cells. Adherent junctions play a role in the stabilization of cell-cell contacts and consist of E-cadherin, nectin as a transmembrane protein, and catenin-associated cytoplasmic protein, which directly connect the structure to the actin cytoskeleton (19). Desmosomes have a significant role in joining adjacent cells to each other and providing anchoring sites for intermediate filaments (20–22). Tight junctions are responsible for water, certain small molecules, and ions passing through the barrier. Tight junction proteins regulate this complex system. These proteins consist of transmembrane (occludin, claudin, junctional adhesion molecules, and tricellulin) and cytoplasmic proteins (zonula occludens, cingulin, and afadin), which help coordinate the alteration of the cytoskeleton (23). Claudin and occludin regulate permeability together with other members of the complex. In human, the epithelial claudin family has 26 members with different functions. Tight claudins are responsible for sealing the intestinal barrier (e.g., types 1, 3, 4, 5, or 6), whereas other claudins can form a pore to precipitate paracellular water and ion transport mechanisms (e.g., types 2 and 15). Certain proteins have been found to have other properties; junctional adhesion molecule A is also considered to be responsible for leukocyte migration (24, 25). Mucosal damage, such as in ulcers of patients with inflammatory bowel disease (IBD), disrupts this complex regulatory structure and causes an uncontrolled leakage through the barrier (26–28) (Figure 1).

Increased permeability of the IEB and dysbacteriosis is considered to be strongly correlated. There is a bidirectional link between the two phenomena: an increase in permeability promotes dysbacteriosis (29), and the changes in the microbiota can also modify intestinal permeability (30, 31). Increased epithelial tight junction permeability promotes commensal bacteria to cause an intestinal cluster of differentiation 4^+^ (CD4^+^) T-cell expansion and interleukin 17A production that limits enteric pathogen invasion (32). In contrast, chronic *Salmonella typhimurium* infection is more severe in transgenic mice with increased intestinal permeability, suggesting that barrier defects ultimately result in enhanced disease progression despite the activation of protective mucosal immunity (32, 33). Altered gut permeability is observed in patients with IBD and even in their first-degree relatives, who are otherwise healthy (34).

Intestinal mucus is a critical component of the IEB since it forms a direct link between the host and the microbiota. The mucous layer comprises a hydrated network of polymers including the mucin glycosylated protein. Mucin consists of a protein core of the proline–threonine–serine (PTS) sequences with tandem repeats and serine and threonine are extensively *O*-glycosylated; this molecular structure forms a “bottle-brush” structure of conformation (35). Glycated mucin domains have water-binding abilities. Mucins exist in two forms: transmembrane mucins are linked to the surface, and secreted mucins form a 3-dimensional network in the intestinal lumen. Membrane-linked mucins have an impact on the composition of the microbiota. The communication between the luminal content and the barrier is supposed to be through specific cleavage, glycosylation, phosphorylation, and some other ways. However, the complete functions of the transmembrane mucins have not yet been discovered completely (35). Mucins are secreted by the goblet cells (36) in the crypts; the main type of secreted mucin is encoded by the Muc2 gene (37, 38), which forms various structures depending on the intestinal segment. Small intestinal mucin is less dense and penetrable to bacteria. Antimicrobial peptides are secreted by Paneth cells, and the passage can help maintain barrier defense. In the large intestine, mucus forms two layers: a dense mucous layer in the proximity of epithelial cells, which is normally impenetrable to bacteria, and the loose mucous layer, which is colonized by commensals. Degradation of the mucus maintains a continuous turnover from dense to loose mucus (39). This layer is an important interface between the luminal microbiota and the epithelial cells, which can maintain intestinal permeability and serve as a nest for the microbiota.

### Microbial Impact on the Innate and Adaptive Immune Responses

During the past two centuries, the incidence of several diseases with multifactorial etiology has increased. In developed countries, there are numerous hypotheses about the pathophysiological background of frequent diseases, such as obesity, asthma, cancer, autoimmune diseases, and allergy. In parallel, developing multidisciplinary molecular biological techniques (“omics”) try to investigate the background of these diseases using microbial, immunological, environmental, and genetic approaches (40, 41).

Immunological examinations have found that the mucosal immune system has a crucial role in the regulation of gut microbiotic homeostasis through optimizing normal and dysbiotic microbiome balance (42). Several pathways seem to be responsible for avoiding dysbiosis. Nucleotide-binding oligomerization domain-containing protein 1 (NOD1) recognizes peptidoglycans from the bacterial wall and suppresses commensal bacteria extension, such as Enterobacteriaceae, Clostridiales, and *Bacteroides* spp. (43). The function of NODs has been proved in transgenic mice studies. Nod2^–/–^ mice have the same commensal microbiota characteristics, and an increased burden of mucosa-associated bacteria leads to mucosal inflammation and colorectal cancer (44–46). In human studies, NOD2 polymorphism has also been connected to Crohn’s disease (CD) (47). Toll-like receptors (TLRs) are responsible for activating the innate immune system *via* sensing microbial particles such as flagellin. Flagellin sensor TLR5 mice^–/–^ have an altered microbial composition compared to the wild type, manifesting in hyperphagia and metabolic syndrome phenotype (48).

Aside from the innate immune system, adaptive changes may also have a significant impact on the regulation of the gut microbiota composition (49, 50). Mucosal IgA, secreted by B cells, binds to a specific bacterium or bacterial particles resulting in neutralization. Human studies have revealed the proximal-distal colonization characteristics of the mucosa (51). Follicular helper T-cells (TFH) promote the secretion of IgA and express programmed cell death protein 1 (PD-1) as well. PD-1 deficiency may then result in an altered microbial composition characterized by increased Enterobacteriaceae and decreased Bifidobacterium abundances (52).

### Microbiome Metabolomics – A New Key for Permeability Regulation and Disease Pathogenesis

Bacterial metabolic products are in the focus of recent studies, including but not limited to the following: short-chain fatty acid (SCFA) metabolites, tryptophan metabolites (serotonin and tryptamine), bacterial lipopolysaccharides (LPS), and peptidoglycans. SCFA metabolites, such as acetate, propionate, and butyrate, can act on fatty acid receptors in epithelial, enteroendocrine, and neuronal cells, and they could affect the central nervous system in experimental model organisms (53, 54). Enterochromaffin cells produce serotonin after SCFA recognition. Neuronal and glial cells react to the fatty acid receptor stimulation in the peripheral nervous system, and potentially, in the central nervous system (55, 56). Tryptophan metabolism is considered to play an extremely important role in gut physiology. Serotonin, a tryptophan derivate with neurotransmitter functions, plays a role in the gut-neuronal/enterochromaffin cells and in gut-bacterial interactions. Another tryptophan metabolic pathway is supposed to be through kynurenine. About 90% of the tryptophan is metabolized in this way. Some of the regulating enzymes are produced in all tissues, and the activation of these enzymes is dependent on the inflammatory cytokines and glucocorticoids. The downstream metabolites of the kynurenine metabolic pathway, quinolinic and kynurenic acids, are neuroactive metabolites acting on the glutamatergic *N*-metyl-D-aspartate (NMDA) receptor (57). Members of the microbiota can act on these enzymes of the cascade. Lactobacilli may accelerate the conversion of tryptophan to kynurenine, which has been linked to behavioral changes in rodent models (58). This finding also demonstrates the functioning of the microbiome-gut-brain axis.

## Diseases Linked to Dysbiosis and Increased Intestine Permeability

Recently, some studies have demonstrated that several diseases are correlated with the perturbation of microbiota and intestinal permeability changes. However, the pathophysiological role of dysbiosis has not exactly been determined yet in the case of several diseases. A short summary is provided on the diseases related to dysbiosis and showed increased intestinal permeability according to the available literature.

### Irritable Bowel Syndrome

Functional gastrointestinal disorders (FGID) are multifactorial diseases with poorly characterized pathophysiology. Several studies have examined the background of IBS, which has a significant impact on the healthcare system due to the high costs of its care. There are regional differences in the prevalence; nonetheless, it involves 12% of the population on average (59, 60). One of the first pathophysiological observations was the increased intestinal permeability in all subtypes of the disease (61–63) seen mostly in the diarrhea-predominant IBS (IBS-D) subgroup (64, 65). Permeability changes have not been verified yet in the constipation-predominant IBS (IBS-C) subgroup by other investigations, whereas patients with mixed bowel habits (IBS-M) have not been investigated so far. In addition, increased permeability correlated with symptom severity in IBS-D (66). It is also well-known that visceral hypersensitivity and dysbacteriosis are important parts of the disease (67).

Nowadays, there is an increasing scientific interest in other gut microbiota components. Fungi are also altered in IBS compared to healthy people, with enrichment of Saccharomycetes and Candida spp., with a distinct genotypic profile and different phenotypical features. However, the significance of these mycobiotic changes has not completely been described. A better understanding of the role and function of archaebacteria, viruses, phages, and protozoa could also change the scientific view of the microbiota in the future (68, 69).

Irritable bowel syndrome pathophysiology studies draw attention to the importance of bacteria through fecal transplantation experiments from patients with IBS-C and IBS-D to germ-free (GF) animals. This intervention could transfer intestinal permeability and visceral sensitivity and transit alterations to the recipient rodents (70–72). Bacterial imbalance is also observed in many patients of IBS; however, the bacterial genera have been found to be very heterogeneous in several studies (73, 74). A Swedish study could not identify any specific microbiota profile in patients with IBS. Nevertheless, microbiome-richness has been found to be lower in the IBS group compared to healthy individuals (75). A greater diversity has been identified to be associated with fewer IBS-like complaints (74). Some studies have found that either Streptococci (73) or Alistipes (76) may be responsible for the IBS symptoms. Nevertheless, no further studies have proved these results. It suggests that the disease pathogenesis is not directly linked to certain phyla, and thus, patients could not be diagnosed exclusively based on the microbiota profile. Novel results in microbiome studies suggest that microbial metabolic products are common factors that determine health or disease conditions (77). A recent study has described that the separation between patients with IBS-D and healthy controls is possible by using proton (1H) nuclear magnetic resonance (NMR) to examine fecal microbial metabolites. Among the 55 metabolites identified, the authors have found five potential biomarkers of IBS-D to distinguish from healthy controls: cadaverine, putrescine, threonine, tryptophan, and phenylalanine (78). Microbial metabolite analysis presents great challenges for future IBS research. Although urine, stool, or volatile samples can be collected in a non-invasive way, the time of elimination has an impact on the degradation of the microbial products, which may have an impact on the analysis (79). It is estimated that more than 50,000 metabolites are present from the microbial metabolism of food, and more than 25,000 compounds can be present in the diet (80). It poses a great challenge for scientists to clearly see through this metabolic jungle in the future.

As mentioned above, serotonin signaling is a potential therapeutic target in IBS (53). SCFA can stimulate serotonin production in the gut from nutrient tryptophan, an essential amino acid. Serotonin production is linked to commensal bacteria through enterochromaffin cells or by direct bacterial production. Bacteria can have an influence on the serotonin metabolism through the regulation of the bioavailability of tryptophan (54–56). Tryptamine is also synthesized from tryptophan by the bacterial tryptophan decarboxylase enzyme. Tryptamine can act on the 5-HT4R serotonin receptor in gastrointestinal motility and secretion (81).

Bacterial LPS and peptidoglycans are components of the bacterial cell wall. They can be recognized by the pattern recognition receptors and can activate and modulate the innate immune system. Mast cell activation and degranulation are followed by immune activation, for instance, the recognition of LPS by toll-like receptors or by IgE receptor activation. Degranulation products, such as histamine, cytokines, chemokines, proteases, and nitric-oxide, have further immune and defensive roles. Histamine takes part in the pathophysiology of IBS and increases intestinal permeability and visceral hypersensitivity (82).

The microbiota can communicate *via* the brain-gut axis and the host immune system through their enzymes and cell wall components. In IBS, this equilibrium is disturbed, as dysbacteriosis, increased gut permeability, visceral hypersensitivity, and mucosal microinflammation are present. The initiator of the pathophysiological process has not been elucidated yet, although acute gastrointestinal infections have been found to initiate the symptoms in a certain group of patients with IBS (post-infectious IBS) (81).

### Inflammatory Bowel Diseases

Inflammatory bowel disease has multifactorial etiology; hence, genetic, environmental, and microbial factors take part in the etiology of the disease. Increased intestinal permeability can be present in remission, while increased intestinal permeability correlated with the severity of the disease in patients with IBD (57, 58, 83, 84). Increased permeability without severe mucosal damage is caused by tight junction protein abnormalities, whereas severe mucosal damage disrupts the barrier and causes uncontrolled leakage of the luminal contents. Dysbacteriosis is also documented in patients with IBD. Reduced bacterial diversity, decreased relative abundance of Firmicutes, and an increase in the number of Proteobacteria are the common patterns described in these studies. In ulcerative colitis, alterations of Roseburia are present, and *Faecalibacterium prausnitzii* appears to be particularly underrepresented. Geographical variations of the disease were also observed. In Chinese patients with ulcerative colitis, the presence of Gardnerella and, in patients with colonic CD, the presence of Fusobacterium have been found to be important (85–87). Some studies point out the role of the bacteriophages and the virome in IBD pathogenesis, although this field has not been well studied yet (88, 89). The role of the intestinal mycobiota is under investigation, and the importance of the fungal fraction of the gut can be demonstrated in IBD by a CD biomarker ASCA antibody (anti-*Saccharomyces cerevisiae*). The metabolomic approach to the gut microbiome suggests that the diminished SCFA level caused by the lower abundance of Firmicutes and similar bacteria may have an effect on the immune system. The determination of the significance and the therapeutic role of SCFA in IBD needs further investigation. In general, tryptophan metabolism is also impaired in IBD. Tryptophan deficiency aggravates disease severity (90), and indole also has a role in the maintenance of gut health (91). Enzymes involved in tryptophan metabolism are studied as potential therapeutic targets. Whether dysbiosis is a cause or a consequence of IBD has not been determined yet. Nevertheless, pathogenic bacteria can invade the mucosa in IBD.

Future IBD diagnostic tools are proposed using microbiota analysis. The latest studies have identified bacterial markers obtained from Campylobacter spp. indicating disease activity in CD (92). The presence of Faecalibacterium is a sign of successful ustekinumab therapy in anti-tumor necrosis factor-alpha (anti-TNF-α) refractory patients with CD (93).

A better understanding of the microbiota–gut interaction in IBD will be helpful in developing novel therapeutic and diagnostic options in the future.

### Chronic Liver Diseases

Morbidity and mortality of chronic liver diseases (CLD) increase rapidly worldwide. The most common causes of CLD are chronic alcohol abuse, Hepatitis B and C virus infection, and non-alcoholic fatty liver disease (NAFLD). CLD can provoke the weakening of mucosal immunity.

Changes in the microbiota composition are observed in pre-cirrhotic patients with CLD, including the reduction of the diversity and overgrowth of the potentially pathogenic Enterobacteriaceae and Enterococcaceae. In viral hepatitis, modified fecal microbiota can be observed before the appearance of the cirrhotic stage (94). A human study has classified patients into NAFLD, control, and healthy donor groups based on their liver biopsy and analyzed fecal samples of the subjects. The NFALD group was then divided into two subgroups: patients with simple steatosis and steatohepatitis. The authors have verified a lower relative abundance of Bacteroidetes in the steatohepatitis group. This observation is independent of body mass index and fat intake (95). A similar microbiota profile has been observed in Hepatitis B virus-induced cirrhosis. Reduced intestinal blood perfusion, mesenteric ischemia, and decreased bowel movements caused by cirrhosis have appeared to change the normal microenvironment to be less suitable for beneficial populations of *Bacteroides* and *Clostridium*, resulting in invasion and colonization of opportunistic pathogens, such as Enterobacteriaceae and *Veillonella*.

*Veillonella* can hydrolyze conjugated bile salts and promote the impairment of micelle formation resulting in cirrhosis. Bile salt hydrolases (BSHs) are members of the Choloylglycine hydrolase family and are important in bile acid metabolism and deconjugated bile acid formation. BSHs have been isolated from several species of intestinal bacteria, mostly by *Bacteroides* and *Clostridium*. Under normal conditions, intestinal anaerobic microbiota cannot metabolize glutathione. In the case of viral hepatitis, the ability of the intestinal microbiome to metabolize glutathione helps the body in detoxification, while bacterial generation of glucose from non-carbohydrate carbon substrates, such as pyruvate, helps to maintain the body’s energy supply. Branched-chain amino acids, nitrogen and lipid metabolism acceleration and decreased aromatic amino acid levels, and cell cycle-related metabolism have been observed in the microbiota of patients with viral hepatitis (96).

Alcohol consumption causes mast cell degranulation and endotoxemia, and it increases intestinal permeability. This effect depends on the degradation of ethanol to acetaldehyde by the microbiota, which can be antagonized by antibiotic treatment (97). In alcoholic liver diseases, the role of the gut-brain axis should also be taken into account since anxiety and depression are usually coupled with excessive alcohol consumption (98). The latest observations show that colonization of Streptococci can predict liver damage in alcoholic liver disease (99).

The incidence of NAFLD is increasing in western countries. The common co-morbidity with obesity, diabetes, and metabolic syndrome makes it almost impossible to distinguish the pathophysiology (100). In these patients, an increase in the Enterobacteriaceae abundance is associated with endotoxemia (101). Some recent data suggest that the altered gut microbiome can produce alcohol in the intestinal lumen, which in turn is responsible for liver damage just like in direct alcohol consumption (102, 103).

Hepatic encephalopathy, as a consequence of advanced liver disease, is the major manifestation of the perturbed brain-gut microbiome axis. Experiments on cirrhotic GF mice and conventional mice have shown that ammonia level in cirrhotic animals is higher than in non-cirrhotic GF ones and similarly in conventional mice, but neuro-inflammation and microglial activation have only been seen in the conventional cirrhotic mice (104). Microbial imbalance is responsible for neurological symptoms. Recent studies have demonstrated promising results concerning the safety and efficacy of fecal microbiota transplantation in the treatment of hepatic encephalopathy (105). However, further research is needed before the clinical application of this treatment.

### Obesity, Chronic Kidney Disease, and Cardiovascular Diseases

Obesity has an increasing prevalence, estimated to be 42% in the United States in 2017–2018 (106). Patients with obesity have altered gut microbiome, as food and fiber intake can be a driver of natural selection in the gut microbiome. Studies with GF and conventional mice have described that GF animals have lower body weight and less white adipose tissue than the conventional ones despite the increased calorie intake (107, 108). Furthermore, GF mice have increased insulin sensitivity and accelerated cholesterol metabolism compared to conventional mice (109). Fecal microbiota transplantation derived from humans with obesity to GF mice caused excessive weight gain compared to lean ones (110). Similar observations have been made with humans after fecal microbiota transplantation (111, 112). These findings underline the causative role of the microbiome in weight and metabolic changes. LPS are important regulators of these diseases, as long-term LPS administration to mice has induced weight gain, insulin resistance, and increased intestinal permeability (113). Short intravenous administration of LPS in humans has induced a storm of cardiovascular hormonal and cytokine markers (113), suggesting that the bacteria increase the cardiovascular risk. There is a link between bacterial metabolism, dietary choline intake, and cardiovascular risk (114). The food sources for phosphatidyl-choline (lecithin) might be eggs, milk, liver, red meat, poultry, shellfish, and fish. The intestinal microbiota can metabolize phosphatidylcholine to trimethylamine. This metabolite is the substrate for the hepatic flavin monooxygenase enzyme, which forms trimethylamine N-oxide. This metabolite is responsible for the regulation of the surface expression levels of macrophage scavenger receptors known to participate in the atherosclerotic process. In a study, the blood microbiome has been analyzed by comparing the samples of patients at high cardiovascular risk but free of coronary disease and the samples of patients who had myocardial infarction (107). An increase in blood bacterial DNA concentration has been observed, which was dependent on blood low-density lipoprotein cholesterol elevation in the myocardial infarction group. Differences in the proportion of numerous bacterial taxa in blood have been significantly modified with the onset of the myocardial infarction. Some of the bacteria, the proportions of which are decreased in patients with myocardial infarction, are known to include species that can metabolize cholesterol. Further research would be promising to find microbiology-based biomarkers for the diagnosis or treatment.

Kidney diseases could also be modulated by the microbiome. Calcium-nephrolithiasis is caused by calcium oxalate stone formation. Oxalate excretion results from endogenous catabolism of hydroxyproline, uracil, orotic and ascorbic acids, and oxalate can originate from dietary sources. The presence of oxalate-degrading functionality in the gut microbiota may limit oxalate absorption and reduce oxalate excretion (115) and oxalate degrading microbiota function can reduce oxalate absorption and excretion. Gram-negative gut commensal *Oxalobacter formigenes* was able to degrade oxalate in the intestinal lumen (116). This bacterium has high oxalate degrading activity; however, probiotic supplementation of *O. formigenes* has not had an impact on the course of nephrolithiasis or oxalate excretion. A recent study has revealed several bacteria with a lesser extent of oxalate degrading activity compared to *O. formigenes*. The analysis has demonstrated an increased representation of these taxa in the fecal samples of non-lithogenic subjects. The complex intestinal metabolic synergy may help maintain the oxalate metabolism (117).

Chronic kidney disease-microbiome interaction has become the focus of interest over the past decade. The alteration of the microbiome is observed in these patients (118, 119). Normal gut microbiota-derived SCFAs stimulate glucagon-like peptide-1 secretion, which exerts protective effects against renal oxidative stress and chronic hyperglycemia. In renal failure, perturbed intestinal microorganisms produce several metabolic products that can have an influence on the kidneys, including indoxyl sulfate, trimethylamine N-oxide, phenylacetylglutamine, and p-cresyl sulfate. Bacterial tryptophan metabolism metabolites are considered to take part in the development of hypertension and chronic kidney failure (120, 121). Uremic toxins of bacterial origin are large, protein-bound toxins, which cannot be eliminated by hemodialysis. Advanced glycation end products lead to oxidative stress and inflammation. Their effect is more general through the receptors for advanced glycation end products (RAGE), which are concentrated in the heart, lungs, and skeletal muscles. Advanced glycation end products finally lead to arterial stiffness, diabetic nephropathy, and endothelial dysfunction. Patients with kidney disease are characterized by congestion in the intestinal tract, reduced fiber digestion, and metabolic acidosis. Each of these factors take part in the increase of intestinal permeability (122). An important component of kidney failure pathogenesis is bacterial hydrolysis of urea by ureases within the GI tract. This reaction leads to increased gut luminal ammonia and increased intestinal pH. The changes in the intestinal microenvironment aggravate dysbacteriosis (123). Higher gut pH induces the expression of tryptophanase (124). Tryptophanase limits tryptophan availability to the host, which influences serotonin levels affecting the enteric and central nervous systems (125–127).

### The Gut–Lung Axis

Lung microbiota has recently got into the focus of scientific interest. Most of the studies focus on the bacterial component (128), but the fungal, viral, and other parts of the ecosystem are almost neglected (129). Lung microbiota is less prominent in terms of quantity than the gastrointestinal microbiota; however, it is originally colonized by the oropharynx and microaspirations from the gastrointestinal tract. The predominant bacterial phyla in the lungs and the gut are identical, and mainly Firmicutes and Bacteroidetes have been observed (130). The fungal component is also prominent, which communicates with the bacteria. Intestinal and lung microbiota are in parallel throughout life, although changes in diet affect not only the intestinal microbiota but also the lung microbiome (131, 132). The bidirectional crosstalk has been demonstrated by animal experiments. It has been proven that LPS instillation in the mouse lungs is resulting in bacterial changes in the gut thereafter (133). The communication between the lungs and the gut is not just the direct link through aspiration and immune modulation, but a so-called co-immunity can play the most important role in the gut–lung axis. Intestinal microbiome members induce immune tolerance and block pathogen colonization through the activation of the immune system and the direct and indirect actions of the microbiota. When the immune system “learns” to recognize the enemy from the microbiome, the effect may also occur in a distant organ (129, 134). This dynamic interaction is now in the focus of studies on chronic lung diseases.

### Cancer and the Gut Microbiome

It is also known that microbiota can play a role in tumorigenesis. Yet, this field of research is still in an early stage. Several observations have been made in different types of cancer to find the microbial key of the disease. The importance of *Helicobacter pylori* in the development of gastric cancer is now widely accepted, and the eradication of this bacterium reduces the risk of gastric cancer (135). Although the pathogenetic steps have not completely been explored, nowadays, research has highlighted the role of the intestinal microbiota in *H. pylori-*mediated gastric cancer development. The intestinal microbiota colonization produces inflammatory metabolites, which establishes the way for carcinogenesis. A study has found that Lactobacilli and Fusobacteria colonize the stomach in gastric cancer. Intestinal microbiota colonization is considered an important step in the pathogenesis of stomach cancer. Microbial metabolites have an effect on inflammation and carcinogenesis (136). *H. pylori* can regulate several signaling pathways, stimulate inflammation and immune responses, and trigger epithelial atrophy, achlorhydria, and dysplasia in cancer (137, 138).

An increase in the relative abundance of *Fusobacterium nucleatum* has been observed in fecal samples of patients with colorectal cancer compared to healthy humans (139). Perturbations in the gut microbiota expose the intestine to inflammatory and genotoxic metabolites such as secondary bile salts, trimethylamine N-oxide, hydrogen sulfide, heme, nitrosamines, heterocyclic amines, and polyaromatic hydrocarbons. The production of these metabolites is augmented due to dietary factors, such as red or processed meat, and a diet poor in fibers (140). These carcinogenic metabolites are called oncotoxins (141). Indigestible dietary fibers in the intestine are metabolized by the gut microbiome into SCFAs such as acetate, propionate, and butyrate, and they have an anti-inflammatory effect on the colonic mucosa. These products are considered to be protective against colorectal cancer. It is still not known whether dysbacteriosis is the cause or the consequence of diet-induced inflammation. Dysbiosis alone is not sufficient for tumorigenesis, but genetic and environmental factors are needed for cancer progression. Certain bacteria have been found to be abundant in colorectal cancer. *F. nucleatum* promotes myeloid infiltration of intestinal tumors in ApcMin/^+^ mice, and it increases the expression of pro-inflammatory genes (142). *F. nucleatum* upregulates inflammatory factors and microRNA 21 through toll-like receptors and causes the activation of the mitogen-activated protein kinase cascade (143). Pancreas cancer tissue can be colonized by the intestinal commensal Gammaproteobacteria. These bacteria can metabolize gemcitabine through their specific enzymes, leading to the diminished effect of chemotherapy (144). The microbiome can also have an additive effect on oncotherapy. The efficacy of the checkpoint inhibitor immunotherapy is augmented in the presence of Bifidobacterium and *Bacillus fragilis* through the shaping of the host immune system (145, 146). A better understanding of the microbiome-immune system interactions will help us develop more targeted, specific antitumor medications in the future.

### Neuropsychiatric Disorders and the Gut-Brain Axis

The microbiome–gut-brain axis does not only affect the gastrointestinal system, but it has a role in the development of various behavioral and neurodegenerative diseases. The relevance of the microbiota in the development of the central nervous system has been well demonstrated by previous studies using GF animals. GF mice present behavior changes such as hyperactivity and memory and learning deficits. GF mice have alterations in 5-HT1A serotonin receptor expression and NMDA receptor in the hippocampus, changes in myelination in the prefrontal cortex, and impairment of the blood-brain barrier (56, 147–150). For the understanding of neuropsychiatric disorders, it is important to know that neurogenesis in the cortex is a prenatal process, while gliogenesis happens both prenatally and after birth; thus, the intrauterine and early life periods should have importance in the development of these diseases.

Autism spectrum disorder is one of the first extraintestinal diseases in the research of the brain-gut axis where increased intestinal permeability has been observed (151). Autism is considered to be the consequence of defective neuronal development. Several genetic factors have been discovered; however, none of them explain the pathogenesis of the disease universally (152). The causative role of increased intestinal permeability and microbiota imbalance has been the focus of research interest since gastrointestinal symptoms are very common in autism spectrum disorder. Fecal samples of autistic children contain a significantly higher proportion of Clostridia and Ruminococci and a lower relative abundance of *Akkermansia muciniphila* compared to healthy populations. The lower bacterial mucinolytic activity is supposed to contribute to the slower mucus turnover and to the “leaky gut,” leading to intestinal inflammation. One recent animal study has hypothesized that maternal salt intake might be responsible for autism. High salt diet-induced dysbacteriosis in mice, and the offspring had also dysbacteriosis coupled with behavior alterations. However, dietary interventions that are effective in patients with IBS or other probiotic supplementation have not shown the expected effect in the case of autism spectrum disorder. We can assume that these abnormalities initiate a cascade effect during the period of neuronal or glial development, which has a permanent effect on the behavior. In the symptomatic phase, the treatment of gut leakiness does not radically change the behavior (153).

Anxiety disorder, major depression, bipolar depression, and schizophrenia are also linked to the microbiome-gut-brain axis. Anxiety disorder and depression are often observed in patients with IBS. Microbiota imbalance is also documented in these diseases, but research in this field is in an early stage (154).

In neurodegenerative diseases, including Alzheimer’s disease and Parkinson’s disease, studies have detected gut microbial imbalance (155, 156). In the case of Alzheimer’s disease, the amyloid-β peptide accumulation is considered to be the hallmark pathology. A study in mice has demonstrated that this peptide has antimicrobial effects. As a part of the immune system, it has a protective role for infections, and it is a double-edged sword in the brain (157). This observation suggests an antimicrobial activation of the immune response, which is linked to dysbiosis. In Parkinson’s disease, gut dysbacteriosis and gastrointestinal dysfunctions can precede the neurological symptoms, suggesting a pathophysiological role of the intestine. Toll-like receptor misrecognition is considered to be an important step in alpha-synucleinopathy progression, and increased intestinal permeability, immune activation, and enteric neuroglial activation are keys in the pathophysiology of Parkinson’s disease (158). Recent studies focus on early signs of the disease and therapeutic targets in the intestine.

As discussed in the previous paragraphs, the spectrum of the diseases in which the brain-gut–microbiome axis is known to play a role is increasing. However, it is currently unclear about which of the diseases will be modifiable through the intestinal microbiota. Most of the modifications of the microbiota are not targeted now and not disease-selected or individual-selected. Fecal microbiota transplantation is also at the center of research interest, but the key questions are to find the indication spectrum and donor selection.

### The Gut–Bone Axis

**FIGURE 2:**
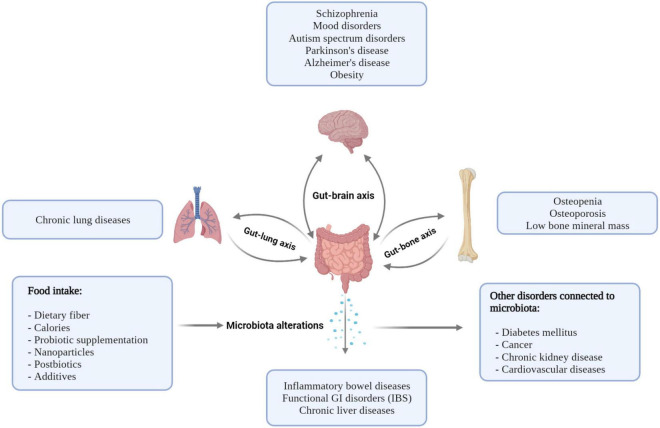
Possible connections between the intestinal microbiota and various organs result in various disorders. Nutrition and diseases have an impact on the intestinal microbiome, and the microbiome has a potential role in the pathogenesis of disorders.

Recently published studies have verified the connection between the gut microbiota and bone metabolism not only in ill, but also in healthy, subjects (159). Some authors have claimed that microbiota alterations have an effect on osteoclast–osteoblast activation and skeletal homeostasis regulation *via* nutritional and immunological pathways and also through bacterial metabolites. Increased abundance of Lactobacillus, Actinomyces, and Blautia has a correlation with osteoporosis (OP) compared to patients with normal bone mass density (BMD) (43). There are some nutritional effects throughout the gut microbiota with an impact on bone health. After the administration of prebiotic or probiotic products, enhanced mineralization has been seen beside the increased availability of magnesium and phosphorus in rats (160). Microbial metabolites also have an impact on bone metabolism. It has been observed that serum trimethylamine N-oxide (TMAO) level, which is a microbiota-dependent metabolite, is associated with OP. Microorganisms of the gut might play a key role in the osteoblast–osteoclast balance *via* the activation of the immune system. Recent findings have proven that *F. nucleatum* could increase osteoclast differentiation *via* increased expression of IL-17A and TNF-alpha (161). On the other hand, gastrointestinal commensal bacteria, such as Bacteroides, Lactobacillus, and Bifidobacterium, can facilitate the development of Treg cells and, by this, increase the osteoblast activity (162). Further randomized controlled trials are needed to verify the effect of gut microbiota on bone remodeling. Possible connections between the intestinal microbiota and various organs and disorders are summarized in Figure 2.

## Effects of Nutrition on Intestinal Permeability and the Gut Microbiome: Possible Therapeutic Interventions

Food has an essential effect on the intestinal microbiota community. It may have selective advantages for certain phyla and disadvantages for other ones (163). Knowledge about the role of diet in disease modification has been derived from ancient times. However, the impact of the microbiome is less known. There are dietary differences between the industrialized and non-industrialized parts of the world. An Urbanized diet is characterized by decreased fiber and increased sugar consumption and high meat and fat intake. Descriptive studies have demonstrated the increased microbial diversity in the areas of the world where fiber intake is higher in the general diet. The causative role of fiber in microbial diversity is demonstrated by multiple animal and human dietary intervention studies (164, 165).

Dietary fibers are indigestible carbohydrate polymers, which are substrates of microbial degradation in the intestine. The fermentability, solubility, and viscosity of fibers are key properties that influence their metabolism by the microbiota. Insoluble fibers such as cellulose increase the transit time, which has consequences on the community of gut microbes. Psyllium is also an insoluble fiber but has a higher viscosity, which influences the cholesterol and sugar metabolism of the host. Fermentable fibers that are soluble, such as pectin or β-glucan, are common parts of the diet. Bile acid-binding will change the hosts’ absorption ability and the microbiota microenvironment. Inulin is a soluble, low viscosity fiber, and inulin-type fructans are supposed to have beneficial metabolic effects including bodyweight reduction and the normalization of blood sugar and cholesterol (166). Dietary fibers, which are subjected to bacterial fermentation and which stimulate the growth of certain beneficial microorganisms, are considered prebiotics. SCFAs are important end-products of the fermentation of complex carbohydrates, the key energy sources of enterocytes (167). Various dietary fibers induce the elevation of the relative abundance of different organisms. Further investigations are needed to define the role of the individual dietary fibers on the microbiome, and prebiotic containing food can be an important weapon against these diseases.

Probiotics can be naturally present in the food or they can be added artificially. Kefir and yogurt are natural probiotic products. The health benefits of the regular consumption of food containing probiotics seem to have anti-cancer, anti-inflammatory, metabolism stabilizing, and antihypertensive effects (168). These products have been in the human diet for centuries. Although probiotic supplementation is considered to be safe, long-term probiotic administration in infants or immunocompromised people requires special consideration. Now, it is clear that probiotics in preterm newborns can prevent the development of necrotizing enterocolitis, although the effect could be augmented by the combination of strains and added to prebiotics (169). The universal, industrial addition of probiotics to food could be inappropriate, or it may even be harmful (170).

The long-term consumption of processed food that is rich in meat induces pro-inflammatory modulation in the gut microbiome. This diet is a risk factor for cardiovascular diseases and kidney failure (171, 172). The effect of ultra-processed food is not only the result of the small fiber content, but an excess of certain nutrients and salt may also change the microenvironment of the microbiota. Food additives can also have an additional impact on the microbiota. One of the components can be inorganic nanoparticles. These nanoparticles can pass through paracellular transport, damage the tight junction proteins, activate the immune system, and contribute to disease pathogenesis. Current theories suggest that there is a contribution of nanoparticles in the pathogenesis of some diseases. Though the results are controversial, as iron nanoparticles have no effect on the microbiota, zinc (Zn)-nanoparticles can have some beneficial effects, and other components such as silicon dioxide (SiO_2_) nanoparticles may cause IEB disruption. Titanium nanoparticles are hypothesized to take part in tumorigenesis (173). The real mechanisms of action and the clinical significance of widespread nanoparticle used in the industry are currently being investigated.

Some components are considered protective and are beneficial for gut health. Postbiotics are metabolites of bacterial metabolism that can potentially have beneficial functions (174, 175). Functional food production now focuses on this possible therapeutic approach to promote health.

Specific components of food, for example, ginseng or resveratrol from cabernet sauvignon grape extract, are considered to be healthy through microbial modifications (175, 176). Generally, dietary polyphenols have an influence on lipid metabolism and related metabolic diseases through the prebiotic effect on the selection of beneficial bacteria.

In the case of obesity, dietary regimes are used to reduce body weight. Calorie restrictive diet and intermittent fasting also have an influence on the gut microbiome. Fifteen replications of intermittent fasting with the same calorie intake have induced body weight loss in mice (177). The role of the microbiome is supported by the fact that this metabolic profile has been transmitted to microbiota-depleted mice *via* fecal microbiota transplantation (177). Human studies are still to be conducted to verify similar effects in human.

Xyloglucan is a food component from the tamarind tree extract, which is often used as a gelling agent in the food industry. This plant-derived polymer has a mucus-like molecular structure. The application of xyloglucans on the mucosal surfaces may function as a protective agent in the gut barrier. In the intestine, the effect of xyloglucan has a spotlight in research as it is a commonly used additive. The effect is examined in a xyloglucan, a pea protein, and a tannin-containing medical device, and the first results show symptomatic improvement and reduction in diarrhea. However, studies on the hypothesized permeability change and the microbiota composition are yet to be performed (178).

A future direction in the food industry can be functional food, which shapes the intestinal microbiota to a health-promoting composition. A varied, homemade, fruit- and vegetable-rich Mediterranean diet has similar health-promoting effects on the microbiota (179–181).

## Summary

The microbiota plays a crucial role in the maintenance of human health. While dysbiosis is observed in several diseases, its pathological background has not yet been fully clarified. Increased intestinal permeability can be a decisive step in the pathophysiological effect of dysbiosis. Diet has a huge impact on the microbiota, but the development of direct, individualized dietetic advice or selected microbiota substitutions may restore health in the early stage of the diseases.

## Author Contributions

OI, PB, and TR constructed the manuscript. CK proofread the manuscript. TM read and approved the manuscript and had the final responsibility to submit the study for publication. All authors contributed to the article and approved the submitted version.

## Conflict of Interest

The authors declare that the research was conducted in the absence of any commercial or financial relationships that could be construed as a potential conflict of interest.

## Publisher’s Note

All claims expressed in this article are solely those of the authors and do not necessarily represent those of their affiliated organizations, or those of the publisher, the editors and the reviewers. Any product that may be evaluated in this article, or claim that may be made by its manufacturer, is not guaranteed or endorsed by the publisher.

## Abbreviations

ASCA: anti-*Saccharomyces cerevisiae* antibody
FGID: functional gastrointestinal disorders
GF: germ-free
IEB: intestinal epithelial barrier
IBD: inflammatory bowel disease
IBS: irritable bowel syndrome
IBS-C: constipation-predominant irritable bowel syndrome
IBS-D: diarrhea-predominant irritable bowel syndrome
IBS-M: irritable bowel syndrome with mixed bowel habit
LPS: lipopolysaccharides
SCFA: short-chain fatty acids.
